# A Systematic Analysis of miRNA-mRNA Paired Variations Reveals Widespread miRNA Misregulation in Breast Cancer

**DOI:** 10.1155/2014/291280

**Published:** 2014-05-18

**Authors:** Lei Zhong, Kuixi Zhu, Nana Jin, Deng Wu, Jianguo Zhang, Baoliang Guo, Zhaoqi Yan, Qingyuan Zhang

**Affiliations:** ^1^Department of General Surgery, Second Affiliated Hospital of Harbin Medical University, Harbin 150086, China; ^2^Department of Bioinformatics, Harbin Medical University, Harbin 150081, China; ^3^Department of Internal Medicine, Cancer Hospital Affiliated to Harbin Medical University, Harbin 150040, China

## Abstract

MicroRNAs (miRNAs) are a class of small noncoding RNAs that can regulate gene expression by binding to target mRNAs and induce translation repression or RNA degradation. There have been many studies indicating that both miRNAs and mRNAs display aberrant expression in breast cancer. Previously, most researches into the molecular mechanism of breast cancer examined miRNA expression patterns and mRNA expression patterns separately. In this study, we systematically analysed miRNA-mRNA paired variations (MMPVs), which are miRNA-mRNA pairs whose pattern of regulation can vary in association with biopathological features, such as the oestrogen receptor (ER), TP53 and human epidermal growth factor receptor 2 (HER2) genes, survival time, and breast cancer subtypes. We demonstrated that the existence of MMPVs is general and widespread but that there is a general unbalance in the distribution of MMPVs among the different biopathological features. Furthermore, based on studying MMPVs that are related to multiple biopathological features, we propose a potential crosstalk mechanism between ER and HER2.

## 1. Introduction

MicroRNAs (miRNAs) are a class of naturally occurring small noncoding RNAs. Mature miRNAs are 19- to 25-nucleotide-long molecules that are cleaved from 70- to 100-nucleotide hairpin pre-miRNA precursors [1, 2]. miRNAs regulate the expression of genes and play a vital role in almost every biological process, including cell differentiation, turning signalling pathways on/off, apoptosis, and cell proliferation [2, 3]. Although several models have been proposed for the mechanism underlying miRNA regulation, it is generally accepted that miRNAs regulate gene expression by binding to their target mRNAs [4, 5]. In vertebrate animals, most miRNAs bind to the 3′ untranslated region (3′UTR) of a target mRNA sequence at a partially complementary sequence and induce translation repression or mRNA degradation [6]. Interestingly, a recent study indicated that miRNAs can shift from acting as a repressor to an activator of gene translation during the cell cycle arrest period [7, 8].

Increasing numbers of microRNAs and mRNAs have been found to be related to the development of breast cancer. In contrast to previous studies based only on miRNA or mRNA expression profiles, examining both miRNA and mRNA expression profiles enables us not only to study miRNA and mRNA expression profiles separately but also to examine miRNA-mRNA regulatory pairs together [8–12]. Nevertheless, in many cancer studies based on miRNA and mRNA expression profiles, instead of considering miRNA-mRNA regulatory pairs together, the tendency is to examine either an miRNA or mRNA first and then apply strategies such as computational miRNA target gene prediction algorithms, sequence homology analysis, or expression correlation indexes to identify the corresponding counterpart of the miRNA (mRNA) and, hence, accomplish the integration of the miRNA-mRNA pair [12, 13]. Interestingly, many of these studies share the common assumption that the regulatory relationship between an miRNA and its target mRNAs is negative, and a great deal of research is therefore based on this assumption [8–12]. For example, to identify the target mRNAs of a specific miRNA from hundreds of candidate mRNAs predicted by a computational algorithm, many scientists prefer to choose those mRNAs whose expression is significantly negatively correlated with that of the miRNA. However, this hypothesis of an miRNA negatively regulating its target mRNA conflicts with the results of a recent study showing that, in some cases, miRNAs can activate the translation of their target mRNAs [7, 8]. Moreover, the aberrant expression of miRNAs and mRNAs in breast cancer gives rise to the question of whether the regulatory pattern of miRNA-mRNA pairs varies with the development of this disease [14, 15]. Thus, we attempt to answer this question by studying the possible effects of several breast cancer-related biopathological features on the regulatory pattern of miRNA-mRNA pairs, and we consider the answer to this question to represent the cutting edge of the exploration of the molecular mechanisms of breast cancer.

Here, we propose MMPV as a term that indicates miRNA-mRNA pairs whose pattern of regulation can vary in association with different statuses of biopathological features. We reveal that the distribution of MMPVs is widespread. Moreover, we find that the miRNAs of the MMPVs that are associated with a particular biopathological feature tend to display a significant regulatory effect on the target mRNAs related to a specific status of the biopathological feature and tend to display no significant regulatory effect on the target mRNAs related to different statuses. Furthermore, based on studying MMPVs associated with multiple biopathological features, we propose the existence of a potential crosstalk mechanism between ER and HER2. Importantly, this study demonstrates that the pattern of miRNA-mRNA regulation can be altered in the context of different statuses of biopathological features, and this discovery will benefit further research exploring the molecular mechanisms underlying breast cancer.

## 2. Materials and Methods

### 2.1. miRNA and mRNA Expression Data

Both miRNA and mRNA expression data were obtained from PMID: 21364938 [16]. The data were derived from the expression profiling of 799 miRNAs and 30,981 mRNAs in 101 primary human breast tumours. Five biopathological features of each sample were available. We classified each biopathological feature as showing one of two different statuses: oestrogen receptor positive (ER+)/oestrogen receptor negative (ER−); mutant TP53 (TP53+)/wild type TP53 (TP53−); survival greater than five years (survial5+)/survival less than five years (survival5−); HER2 positive (HER+)/HER2 negative (HER2−); and basal-like breast cancer (basal)/no basal-like breast cancer (nonbasal). The miRNA and mRNA expression data have been submitted to the Gene Expression Omnibus (GEO) under accession numbers GSE19536 and GSE19783, respectively.

### 2.2. miRNA-mRNA Targeting Pairs

We obtained experimentally validated miRNA-mRNA targeting pairs from Tarbase 6.0 [17]. Among the healthy population, the regulatory pattern of 293 miRNA-mRNA pairs indicated positive regulation, while that of 3,628 miRNA-mRNA pairs showed negative regulation.

### 2.3. Computation of the miRNA-mRNA Regulatory Patterns

To examine the regulatory pattern of miRNA-mRNA pairs, which could vary with different statuses of biopathological features, we must quantify the regulatory patterns of the miRNA-mRNA pairs associated with a certain status of a biopathological pattern. For illustrating, here we calculate the regulatory pattern of each miRNA-mRNA pair in ER+ and ER− specimens first. Gene expression with samples in both ER+ and ER− was compiled first, and then the Pearson correlation coefficient (PCC) was adapted to measure the regulatory pattern of miRNA-mRNA pairs associated with a specific status of a biopathological feature. If the PCC is greater than zero, then a positive regulatory pattern corresponds to this PCC and vice versa.

### 2.4. Choosing the miRNA-mRNA Pairs Whose Regulatory Pattern Varies Significantly with Each Biopathological Status to the Normal Condition

Each miRNA-mRNA pair receives 2 PCCs, corresponding to ER+ and ER− statuses, representing its regulatory pattern in ER+ and ER− specimens, respectively. Based on the quantified results regarding the regulatory pattern of each miRNA-mRNA pair, we prefer those miRNA-mRNA pairs whose 2 PCCs showed opposite algebraic signs (sign change pairs) and those whose 2 PCCs showed the same algebraic sign but displayed the ratio of the PCC of each condition to the normal greater than 2 or smaller than 0.5 (fold change pairs). So that our results would have greater biological importance, we later removed miRNA-mRNA pairs whose 2 PCCs were both insignificant (B-H FDR *q* < 0.05).

### 2.5. Representing the Regulatory Patterns of miRNA-mRNA Pairs

Following the two steps described above, we obtained the miRNA-mRNA pairs whose regulatory patterns varied significantly with an ER+ versus ER− status (ER MMPVs). We used the letters U and D to represent positive and negative regulations and ∗ to indicate the significance in the statistic. Thus, given that the regulatory pattern of experimentally validated miRNA-mRNA pairs downloaded from Tarbase 6.0 in the healthy population was known, we used U or D to represent the regulatory pattern of miRNA-mRNA pairs in breast cancer patients with an ER+ or ER− status and in the healthy population. For example, if the regulatory pattern of hsa-miR-1 and CA3 corresponds to negative regulation in the healthy population and to positive regulation in ER+ breast cancer patients and significant positive regulation in ER− breast cancer patients, then we can refer to this pair as D_U_U*. As another example, the regulation of has-mir-375 and FOLR1 is negative in the healthy population, while in ER+ specimens it is positive, whereas it is significantly negative in ER− specimens. Hence, the change in the pattern of regulation can be represented as D_U_D* to indicate the regulatory pattern of hsa-miR-1 and CA3 in the healthy population and in ER+ breast cancer patients and ER− breast cancer patients.

### 2.6. Gene Ontology (GO) Enrichment Analysis of MMPVs

GO database was used to explore the biological function involved in MMPVs. We used Gorilla [18] to conduct GO enrichment analysis, and the *P* value threshold is set as 1.0*E* − 03. The background list comprised all of the genes for the miRNA-mRNA pairs that we obtained from Tarbase 6.0. We placed the mRNAs of each type of MMPV into a target set and obtained the results for the biological process cellular component.

## 3. Results and Discussion

Our method examines miRNA and mRNA gene expression data to obtain MMVPs for five breast cancer-related biopathological features. The statistical summary is shown in Table 1. The definitions of the fold change pairs and sign change pairs were given above.

First, we discuss the general unbalanced enrichment trend in the distribution of MMPVs associated with every type of biopathological feature. Second, we select (B-H FDR *q* < 0.05) MMPVs whose miRNA and mRNA are both significantly differentially expressed between the two statuses of the biopathological features (DE-MMPV), and we check the published literature to confirm their relationship with the corresponding biopathological feature. Third, we analyse MMPVs that are shared by multiple types of biopathological features, and we propose the existence of potential crosstalk between ER and HER2. Fourth, mRNAs of each type of MMPV are analysed for GO enrichment through a hypergeometric gene set enrichment analysis. Finally, we map the mRNAs of the MMPVs to Human Protein Reference Database (HPRD) protein-protein interaction networks to explore the topological features of genes of MMPVs.

### 3.1. The General Unbalanced Distribution of MMPVs

**Figure 1:**
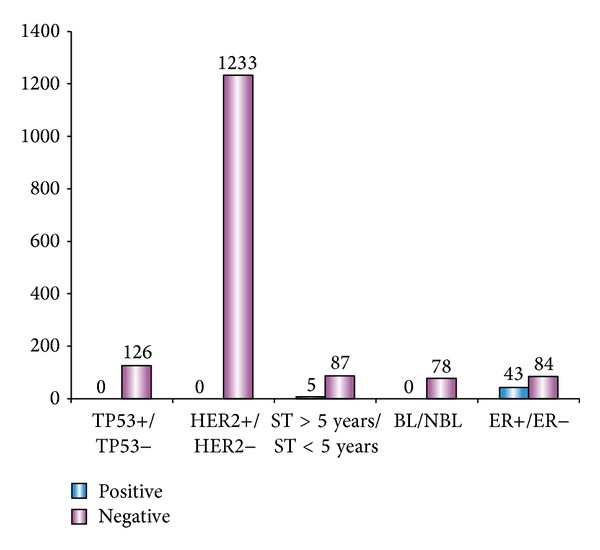
The distribution of MMPVs among the five types of biopathological features. The height of positive bar (in blue colour) represents the number of MMPVs whose regulatory pattern is significant for the first status of a specific biopathological feature, and the height of the negative bar (in red colour) represents the number of MPPVs whose regulatory pattern is significant for the second status of a specific biopathological feature.

Because the number of miRNA-mRNA pairs whose regulatory pattern is positive in the healthy population is relatively small and, hence, cannot reach statistical significance, in this section, we examine only miRNA-mRNA pairs whose regulatory pattern is negative in the healthy population. Their distribution among the five examined types of biopathological features is shown in Figure 1.

For each of the biopathological features, we found that the regulatory pattern of MMPVs tended to be significant for one specific status and to be insignificant for the other status. To be more specific, the miRNAs of the MMPVs tended to have a significant regulatory effect on their target mRNAs for the following statuses: wild type TP53 gene, HER2−, survival time of less than five years, nonbasal-like breast cancer subtype, and ER−.

The above result showed that the overexpression of HER2 is the result of deregulation of genes, rather than gene amplification, and this discovery is consistent with the result of Menard et al. [19]. Considering this result together with our findings, we propose that the widespread decreasing regulatory effect of miRNAs on their target mRNAs contributes to HER2 expression.

Cheng et al. adopted a regulatory effect score (RE score) to evaluate the regulatory effect of miRNAs and discovered that, compared with ER+ patients, most miRNAs exhibit a higher RE score. In other words, they have a more significant regulatory effect on their target mRNAs in ER− patients [20]. This discovery is consistent with our findings.

Suzuki et al. found that three types of missense mutation in the DNA-binding domain of p53 can lead to decreased processing of pri-miRNAs by Drosha [21]. They therefore proposed that p53 mutants might reduce the interaction between pri-miRNAs and Drosha complex proteins and, hence, affect the genesis of mature miRNAs. In this context, mutation of the TP53 gene could decrease the production and activity of miRNAs and ultimately lead to the results that we obtained here: MMPVs related to TP53 tend to exhibit a significant regulatory pattern in a population with the wild TP53 gene.

Most miRNAs can be regarded as antioncogenesis miRNAs given the fact that compared to the healthy population, most miRNAs are downregulated in cancer patients [1]. Moreover, many genes encoding miRNAs are located in regions that are related to cancer, and genes in these regions frequently undergo rearrangement, amplification, and loss [22]. Specifically, genomes associated with basal-like breast cancer tend to be more unstable than those associated with other subtypes of breast cancer [23]. In addition, Blenkiron et al. compared the expression of genes that are involved in the genesis of miRNAs in several breast cancer subtypes and found that Dicer1 was significantly downregulated in basal-like, HER2+, and luminal B cases, all of which are closely associated with poorer prognostic results [24]. Given that basal-like breast cancer patients usually display poorer prognostic results, we propose that compared to nonbasal-like breast cancer, the genome of basal-like breast cancer patients is more unstable, with miRNAs more often being downregulated and gene amplification occurring more frequently, as gene loss and gene rearrangement do. Thus, the production as well as the activity of miRNAs is expected to be lower in basal-like cancer patients, which is consistent with our results.

We did not find any relevant studies that provide any clues about the unbalance in the distribution of the survival time-associated MMPVs. However, we noted that the regulatory patterns of MMPVs related to ER and HER2 status tended to be significant in ER− and HER2− patients. ER− breast cancer patients are usually resistant to Tamoxifen therapy and, thus, show poorer prognostic results [25–27]. Similarly, most HER− breast cancer patients cannot benefit from Trastuzumab therapy, which greatly increases the survival rate in the HER2+ breast cancer patients. Based on the above results, it can be observed that the regulatory pattern of MMPVs tends to be significant in association with statuses that suggest poorer prognostic results. Thus, it is reasonable to propose that the unbalance in the distribution of the survival time-associated MMPVs may result from the unbalance that remains in the distribution of ER- and HER2-related MMPVs. This result reveals the capacity of detecting biologically important regulatory events mediated by miRNAs.

### 3.2. Significantly Differentially Expressed Genes (DEGs) Encoding MMPVs and Their Relationship with Biopathological Features

To explore the relationship between MMPVs and biopathological features, we conducted a significant analysis of microarray (SAM) analysis to detect MMPVs whose miRNAs and mRNAs are both significantly differentially expressed between the two statuses of a given biopathological feature (DE-MMPVs). The final results are shown in Table 2.

First, we analysed the differentially expressed genes (DEGs) among ER-associated DE-MMPVs, and we found that they shared the same miRNA: hsa-miR-375. Pedro de Souza Rocha Simonini reported that hsa-miR-375 is overexpressed in breast cancer tumours with an ER+ status and that decreasing the expression of hsa-miR-375 will decrease the activity of ER accordingly [28]. This observation is consistent with our findings. Thus, we searched the relevant literature to examine five of the target mRNAs of hsa-miR-375. FLOR1 tends to show low expression in ER+ cancers [29]. Signal traducing adaptor protein (STAP2) is regarded as a potential drug target for ER− breast cancer patients because this protein can facilitate the growth of breast cancer cells by interacting with BRK and STAT3/5 [30–33]. STAP2 can also increase the activity of NF-Kb, whose expression is negatively correlated with ER activity [34]. Interestingly, the regulatory patterns of hsa-miR-375 and STAP2 in the healthy population and in ER− and ER+ breast cancer patients are all positive. However, when the expression of hsa-miR-375 is significantly downregulated in ER− specimens, the expression of STAP2 changes, and instead of being downregulated, it is upregulated quite significantly.

Second, we analysed the DEGs among TP53-associated DE-MMPVs. Adan Valladares showed that CCND1 and LAMC1 are overexpressed in breast cancer patients [35]. We observed that, compared to patients with mutated TP53, the expression of CCND1 and LAMC1 is increased in patients with wild type TP53. This observation is of particular interest because it contradicts our expectation that because wild type TP53 inhibits the expression of oncogenes, CCND1 and LAMC1, which are both overexpressed in breast cancer patients, should be downregulated in individuals with wild type TP53.

It can be observed that the regulatory pattern of hsa-let-7b and CCND1 remains positive in breast cancer patients, regardless of the status of TP53, which is negative in the healthy population. Such D_U_U regulatory pattern variation was also found for hsa-miR-145 and MUC1. MUC1 encodes a mucoprotein that is overexpressed in many types of cancer, including breast cancer. Similar to CCND1, MUC1 is a potential biomarker for tumours, and MUC1 plays an important role in the invasion and metastasis of tumours. Specifically, MUC1 can interact with TP53 and inhibit cell apoptosis mediated by TP53, thus facilitating the proliferation of cancer cells [36]. Similar to hsa-let-7b and CCND1, the regulatory pattern of has-miR-145 and MUC1 shifts from being negative in the healthy population to positive in breast cancer patients. No relevant research has shown such an aberrant disturbance of regulatory patterns, and this disturbance is expected to be associated with the genesis and development of breast cancer.

Nguyen et al. reported that hsa-miR-29c negatively regulates DNMT3B, the overexpression of which can cause the hypermethylation of some tumour suppressor genes [37]. Our results show that the regulatory pattern of hsa-miR-29c and DNMT3B is D_D_D in association with TP53. Compared to patients with mutated TP53, the expression of hsa-miR-29c is significantly increased in individuals with wild type TP53, thus enforcing the repression effect on DNMT3B. This observation is supported by the finding of Toledo and Bardot that the wild type P53 protein can bind to the Drosha protein complex and enhance the transcription of tumour suppressor miRNAs [38]. Similar regulatory pattern variation occurs for hsa-miR-107 and CDK6, which tend to be overexpressed in aggressive tumours. We propose that the enhanced regulatory effect of has-miR-107 on CDK6 is also due to the combined action of P53 and Drosha.

Finally, we analysed the DEGs among the subtype-associated DE-MMPVs. We observed that 2 MMPVs (has-miR-145 and MUC1, hsa-miR-155 and CBFB) and one mRNA (CDK6) are shared by the TP53-associated MMPVs and subtype-related MMPVs. Specifically, compared to patients with mutated TP53, the levels of MUC1, CBFB, and CDK6 expression are significantly decreased, significantly increased, and significantly decreased, respectively, in wild type TP53 patients. Interestingly, compared to basal-like breast cancer patients, the levels of MUC1, CBFB, and CDK6 expression are significantly decreased, significantly increased, and significantly decreased in nonbasal-like breast cancer patients, respectively. All of these findings indicate that these three genes could play similar roles in TP53 pathways and biological pathways that are related to basal-like breast cancer. Moreover, we found that, compared to basal-like breast cancer patients, the expression of ETS is significantly decreased in nonbasal-like cancer patients. This finding is supported by the work of Charafe-Jauffret et al., who reported that the expression of ETS1 is higher in basal-like breast cancer than in other breast cancer subtypes [39]. Furthermore, compared to basal-like breast cancer, the levels of CSF1R and CBFB expression significantly decrease in nonbasal-like breast cancer. Furthermore, this finding is reasonable because CSF1R is overexpressed in invasive breast cancer and is strongly associated with a shorter survival time [40], and CBFB is regarded as a potential oncogene [1].

### 3.3. Analysis of MMPVs Associated with Multiple Biopathological Features

We found that many MMPVs are associated with multiple biopathological features. The distribution of these MMPVs is shown in Table 3.

To find genes that are closely associated with two different biopathological features, we further filtered the data that appear in Table 3. For example, to find genes that are related to both ER and TP53 status, we first selected all of the mRNAs in the 31 MMPVs that are associated with both TP53 and ER. Then, we selected the miRNAs present in TP53- and ER-associated MMPVs as well as in MMPVs associated with other biopathological features. Finally, we uncovered two genes (MYBL and M6PRBP1), which are expected to be closely related to ER and HER2.

**Figure 2:**
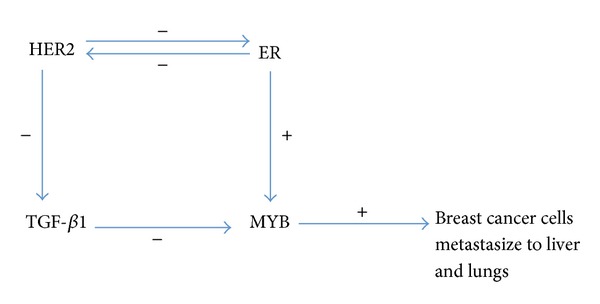
Proposed crosstalk between ER and HER2. Gene ontology (GO) enrichment analysis of MMPVs.

There is a substantial amount of research examining crosstalk between ER and HER2. Isabel Pinhel claimed that the levels of HER2 and ER expression are positively correlated in non-HER2-overexpressing breast cancer tumours and are negatively correlated in HER2-overexpressing breast cancer tumours [41]. HER2 overexpression can repress the antiproliferation effect of TGF-*β*1 and, hence, enhance the growth of cancer cells [42]. Moreover, TGF-*β*1 can repress the expression of MYB, and ER+ status enhances the expression of MYB [43]. Importantly, MYB and MYBL are expected to display similar functions given that these two proteins belong to the same transcription factor family, and they are homologous. It has been reported that MYB is relevant to hematopoietic function [43]. An experiment conducted by Mucenski and colleagues in MYB knockout mice showed that MYB is closely related to hematopoietic function, especially hematopoietic cells in the liver, as all MYB knockout mice ultimately die as a result of hypoxia, and their livers are anaemic and relatively small compared to the livers of mice in the control group [44]. Furthermore, the cancer cells of cancer patients exhibiting HER2 overexpression are more likely to metastasise to the lungs and liver [45]. Considering our findings together with the supporting results from the literature noted above, we propose the existence of potential crosstalk between MYB, overexpressed HER2, and ER as shown in Figure 2.

This proposed potential crosstalk between MYB, overexpressed HER2, and ER not only will contribute to further studies addressing the molecular mechanisms underlying breast cancer but also serves as an important reference for potential joint therapy with tamoxifen and trastuzumab.

We conducted a GO enrichment analysis of the mRNA components of MMPVs. The results of the biological process (BP) enrichment analysis and cellular component (CC) enrichment analysis are shown in Supplementary Table 1 in Supplementary Material available online at http://dx.doi.org/10.1155/2014/291280.

It can be observed that the GO terms related to TP53 mainly include molecules that are involved with cell adhesion to the extracellular matrix. There have been many studies concentrating on the relationship between TP53 and tumour metastasis, which is closely associated with cell adhesion and the extracellular matrix. Specifically, Abramson et al. reported that compared to individuals with wild type TP53, the strength of cell adhesion is greatly increased in the population exhibiting mutant TP53 [46]. Anaganti et al. claimed that wild type TP53 can repress the expression of focal adhesion kinase (FAK), which is a critical regulator of adhesion and motility whose overexpression is strongly associated with enhanced metastatic potential. Additionally, FAK is frequently overexpressed in populations with mutant TP53 [47].

The GO terms related to the ER are mainly associated with DNA synthesis and the cell cycle. S F Doisneau-Sixou claimed that oestrogen independently regulates the expression and function of c-Myc and cyclin D1. Antioestrogen treatment of MCF-7 cells can greatly decrease the expression of c-Myc and cyclin D1, resulting in the arrest of the cell cycle and inhibition of DNA synthesis.

### 3.4. Topological Features of Genes Encoding MMPVs

We explored the topological features of the genes encoding MMPVs by mapping the mRNAs of each type of MMPV to the HPRD protein-protein-interaction (PPI) network [48]. Specifically, we employed Student's *t*-test to compare the average degree of the MMPV genes with those of PPI network. In fact, there are biases which existed in current PPI databases. Here we use the commonly used database, HPRD. The results are shown in Table 4. Except for subtype-associated MMPVs, the average degree of the mRNAs related to all of the other biopathological features is significantly greater than the average degree in the HPRD-PPI network.

*P* values were calculated using a one-tailed *t*-test. The *P* value shown in bold indicates that the average degree of the corresponding biopathological feature is significantly greater than that in the HPRD-PPI network.

## 4. Conclusions

In this study, we discovered that the regulatory pattern of miRNA-mRNA pairs can vary with different statuses of biopathological features. To further explore the molecular mechanisms underlying breast cancer, we studied five biopathological features (the ER, HER2 and TP53 genes, cancer subtype, and survival time) that are closely related to breast cancer. We observed a general unbalance in the distribution of MMPVs. Moreover, the differentially expressed MMPV genes suggest that there is a potential effect of these biopathological features on the development of breast cancer at the molecular level. Furthermore, we examined the topological features of genes encoding MMPVs in the HPRD PPI network, and we propose the existence of potential crosstalk between ER and HER2. The method developed in this paper can help detecting biologically important regulatory events mediated by miRNAs.

## Supplementary Material

Gene ontology (GO) enrichment analysis was done on cellular component and biological processes branches. GO terms related to TP53 mainly include molecule that are involved with cell adhesion to the extracellular matrix.

## Figures and Tables

**Table 1:** Statistical results for the MMPV responses to different features.

Feature	Number of fold change pairs	Number of sign change pairs	Total number of MMPVs
TP53	110	49	159
ER	72	30	102
Her2	516	768	1,284
Survival time	0	0	0

**Table 2:** Distribution of DE-MMPVs associated with ER, TP53, and subtype status.

Feature	miRNA	mRNA	Regulatory pattern
ER	hsa-miR-375(D)	PRKX(U)	D_D_D*
	hsa-miR-375(D)	FOLR1(U)	D_U_D*
	hsa-miR-375(D)	STAP2(U)	U_U_U*
	hsa-miR-375(D)	KIAA0232(D)	U_U_U*
	hsa-miR-375(D)	TBX19(U)	U_D_D*

TP53	hsa-miR-7(D)	ALG3(D)	D_U_U*
	hsa-miR-155(D)	VCAM1(D)	D_U_U*
	hsa-miR-155(D)	ETS1(D)	D_U_U*
	hsa-miR-155(D)	CBFB(D)	D_U_U*
	hsa-miR-155(D)	ARL5B(D)	D_U_U*
	hsa-miR-145(U)	MUC1(U)	D_U_U*
	hsa-let-7b(U)	CCND1(U)	D_U_U*
	hsa-miR-375(U)	LDHB(D)	D_U_D*
	hsa-miR-7(D)	TCOF1(D)	D_D_U*
	hsa-miR-7(D)	KCNJ14(D)	D_D_U*
	hsa-miR-145(U)	FSCN1(D)	D_D_U*
	hsa-let-7b(U)	CHMP2A(U)	D_D_U*
	hsa-miR-375(U)	PRKX(D)	D_D_D*
	hsa-miR-29c(U)	LAMC1(U)	D_D_D*
	hsa-miR-29c(U)	DNMT3B(D)	D_D_D*
	hsa-miR-29c(U)	COL3A1(U)	D_D_D*
	hsa-miR-214(U)	HSPD1(D)	D_D_D*
	hsa-miR-155(D)	ARID2(U)	D_D_D*
	hsa-miR-145(U)	CCDC43(U)	D_D_D*
	hsa-miR-107(U)	CDK6(D)	D_D_D*
	hsa-let-7b(U)	SPCS3(D)	D_D_D*

Subtype	hsa-miR-155(D)	ETS1(D)	D_U_U*
	hsa-miR-155(D)	CSF1R(D)	D_U_U*
	hsa-miR-155(D)	CBFB(D)	D_U_U*
	hsa-miR-146a(D)	SAMD9L(D)	D_U_U*
	hsa-miR-146a(D)	EPSTI1(D)	D_U_U*
	hsa-miR-146a(D)	BCL2A1(D)	D_U_U*
	hsa-miR-145(U)	MUC1(U)	D_U_U*
	hsa-miR-375(U)	AKAP7(D)	D_U_D*
	hsa-miR-193b(U)	MAT2A(U)	D_D_U*
	hsa-miR-145(U)	FSCN1(D)	D_D_U*
	hsa-let-7b(U)	CHMP2A(U)	D_D_U*
	hsa-miR-29c(U)	CDK6(D)	D_D_D*

**Table 3:** Distribution of MPPVs that are associated with multiple pathological features.

Feature 1	Feature 2	Overlap
ER	Survival	3
Subtype	Survival	3
ER	Subtype	4
TP53	Subtype	6
TP53	Survival	6
ER	TP53	11
HER2	Subtype	12
HER2	Survival	14
HER2	TP53	31
HER2	ER	40

**Table 4:** Comparison of the average degree of the different types of MPPVs with that in the HPRD-PPI network.

Feature	Average degree	*P* value
ER	11.76	0.03
TP53	11.23	**0.01**
HER2	11.74	6.38**E** − 08
Survival	12.81	5**E** − 03
Subtype	10.60	0.095
HPRD-PPI network	7.80	
