# Supplementary material for: A Systematic Analysis of miRNA-mRNA Paired Variations Reveals Widespread miRNA Misregulation in Breast Cancer
Source: Biomed Res Int. 2014 May 18;2014:291280. doi: 10.1155/2014/291280 (PMC4052615; doi:10.1155/2014/291280)
Supplement: Supplementary file 1 — Gene ontology (GO) enrichment analysis was done on cellular component and biological processes branches. GO terms related to TP53 mainly include molecule that are involved with cell adhesion to the extracellular matrix. [file 291280.f1.docx]

**Sup. Table 1**. GO enrichment results for cellular components (C) and biological processes (B)

| Feature | ID | P-value | Description |
| --- | --- | --- | --- |
| TP53 | B-GO:0030155 | 2.63E-05 | regulation of cell adhesion |
|  | B-GO:0010810 | 7.75E-05 | regulation of cell-substrate adhesion |
|  | B-GO:0031589 | 3.44E-04 | cell-substrate adhesion |
|  | B-GO:0051385 | 3.69E-04 | response to mineral corticoid stimulus |
|  | B-GO:0070206 | 4.82E-04 | protein trimerisation |
|  | B-GO:0030198 | 5.44E-04 | extracellular matrix organisation |
|  | B-GO:0043062 | 5.44E-04 | extracellular structure organisation |
|  | B-GO:0007159 | 6.37E-04 | leukocyte cell-cell adhesion |
|  | B-GO:0032259 | 9.30E-04 | methylation |
|  | C-GO:0005583 | 4.82E-04 | fibrillar collagen |
|  | C-GO:0031012 | 8.04E-04 | extracellular matrix |
| ER | B-GO:0006271 | 2.42E-07 | DNA strand elongation involved in DNA replication |
|  | B-G0:0022616 | 4.19E-07 | DNA strand elongation |
|  | B-GO:0000084 | 4.46E-07 | S phase of mitotic cell cycle |
|  | B-GO:0051320 | 5.98E-07 | S phase |
|  | B-GO:0022402 | 1.17E-06 | cell cycle process |
|  | B-GO:0006259 | 1.80E-06 | DNA metabolic process |
|  | B-GO:0006260 | 3.31E-06 | DNA replication |
|  | B-GO:0007049 | 4.10E-06 | cell cycle |
|  | B-GO:0000278 | 6.00E-06 | mitotic cell cycle |
|  | B-GO:0022403 | 3.24E-05 | cell cycle phase |
|  | B-GO:0006974 | 4.58E-05 | response to DNA damage stimulus |
|  | B-GO:0006281 | 6.55E-05 | DNA repair |
|  | B-GO:0000075 | 1.89E-04 | cell cycle checkpoint |
|  | B-GO:0000082 | 2.38E-04 | G1/S transition of mitotic cell cycle |
|  | B-GO:0071156 | 5.24E-04 | regulation of cell cycle arrest |
|  | B-GO:0032201 | 6.76E-04 | telomere maintenance via semi-conservative replication |
| HER2 | B-GO:0000077 | 2.12E-05 | DNA damage checkpoint |
|  | B-GO:0045859 | 1.21E-04 | regulation of protein kinase activity |
|  | B-GO:0060255 | 1.26E-04 | regulation of macromolecule metabolic process |
|  | B-GO:0031326 | 1.57E-04 | regulation of cellular biosynthetic process |
|  | B-GO:0031570 | 1.95E-04 | DNA integrity checkpoint |
|  | B-GO:0009889 | 2.03E-04 | regulation of biosynthetic process |
|  | B-GO:0080090 | 2.14E-04 | regulation of primary metabolic process |
|  | B-GO:0031323 | 2.23E-04 | regulation of cellular metabolic process |
|  | B-GO:0051171 | 2.34E-04 | regulation of nitrogen compound metabolic process |
|  | B-GO:0009628 | 2.45E-04 | response to abiotic stimulus |
|  | B-GO:0042632 | 2.71E-04 | modification-dependent macromolecule catabolic process |
|  | B-GO:0019941 | 2.71E-04 | modification-dependent protein catabolic process |
|  | B-GO:0031325 | 2.75E-04 | positive regulation of cellular metabolic process |
|  | B-GO:0051603 | 3.82E-04 | proteolysis involved in cellular protein catabolic process |
|  | B-GO:0007187 | 3.82E-04 | G-protein signalling, coupled to cyclic nucleotide second message |
|  | B-Go:0009893 | 4.04E-04 | positive regulation of metabolic process |
|  | B-GO:0044265 | 4.96E-04 | cellular macromolecule catabolic process |
|  | B-GO:0051318 | 6.11E-04 | G1 phase |
|  | B-GO:0019935 | 6.11E-04 | cyclic-nucleotide-mediated signalling |
|  | B-GO:0006511 | 6.89E-04 | ubiquitin-dependent protein catabolic process |
|  | B-GO:0019219 | 7.41E-04 | regulation of nucleobase-containing compound metabolic process |
|  | B-GO:0044248 | 8.00E-04 | cellular catabolic process |
|  | B-GO:0043549 | 8.11E-04 | regulation of kinase activity |
|  | B-GO:0019933 | 8.52E-04 | cAMP-mediated signalling |
|  | B-GO:2000112 | 8.58E-04 | regulation of cellular macromolecule biosynthetic process |
|  | B-GO:0071900 | 8.79E-04 | regulation of protein serine/threonine kinase activity |
|  | B-GO:0051338 | 9.34E-04 | regulation of transferase activity |
|  | B-GO:1900372 | 9.50E-04 | negative regulation of purine nucleotide biosynthetic process |
|  | B-GO:0045980 | 9.50E-04 | negative regulation of nucleotide metabolic process |
|  | B-GO:0030809 | 9.50E-04 | negative regulation of nucleotide biosynthetic process |
|  | B-GO:0030800 | 9.50E-04 | negative regulation of cyclic nucleotide metabolic process |
|  | B-GO:0030803 | 9.50E-04 | negative regulation of cyclic nucleotide biosynthetic process |
|  | B-GO:0030818 | 9.50E-04 | negative regulation of cAMP biosynthetic process |
|  | B-GO:0030815 | 9.50E-04 | negative regulation of cAMP metabolic process |
|  | B-GO:0009262 | 9.50E-04 | deoxyribonucleotide metabolic process |
|  | B-GO:0031328 | 9.65E-04 | positive regulation of cellular biosynthetic process |
|  | C-GO:0005834 | 9.50E-04 | heterotrimeric G-protein complex |
